# Long-Term Effectiveness of Engineered T7 Phages Armed with Silver Nanoparticles Against *Escherichia coli* Biofilm

**DOI:** 10.2147/IJN.S479960

**Published:** 2024-10-04

**Authors:** Mateusz Szymczak, Piotr Golec

**Affiliations:** 1 Department of Molecular Virology, Institute of Microbiology, Faculty of Biology, University of Warsaw, Warsaw, Poland

**Keywords:** bacteriophage T7, *Escherichia coli*, biofilm, silver nanoparticles

## Abstract

The escalating threat of antibiotic-resistant bacteria, particularly those forming biofilm structures, underscores the urgent need for alternative treatment strategies. Bacteriophages have emerged as promising agents for combating bacterial infections, especially those associated with biofilm formation. However, the efficacy of phage therapy can be limited by the development of bacterial resistance and biofilm regrowth. Interestingly, phages could be combined with other agents, such as metal nanoparticles, to enhance their antibacterial effectiveness. Since the therapeutic strategy of using phages and metal nanoparticles has been developed relatively recently, evaluating its efficacy under various conditions is essential, with a particular focus on the duration of activity. This study tested the hypothesis that a novel approach to combating bacterial biofilms, based on phages armed with silver nanoparticles (AgNPs), would exhibit enhanced activity over an extended period after application. In this work, we investigated the potential of engineered T7 phages armed with AgNPs for eradicating *Escherichia coli* biofilm. We demonstrated that such biomaterial exhibits sustained antimicrobial activity even after prolonged exposure. Compared to phages alone or AgNPs alone, the biomaterial significantly enhances biofilm eradication, particularly after 48 hours of treatment. These findings highlight the potential of synergistic phage-nanoparticle strategies for combatting biofilm-associated infections.

## Graphical Abstract

**Figure:**
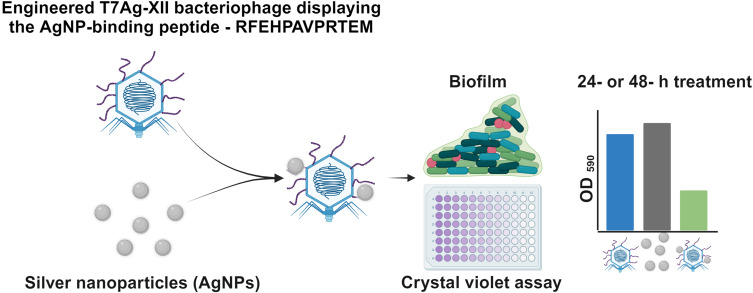

## Introduction

The spread of antibiotic resistance in bacteria poses a danger to public health.^1^ Multidrug-resistant bacteria (MDR), particularly those equipped with biofilm-forming capabilities, are potentially hazardous as they possess the ability to instigate chronic and recurrent infections.^2^ Bacterial biofilm, the complex communities of microorganisms attached to surfaces and encased in an extracellular polymeric substance (EPS) matrix, is notoriously resistant to antibiotics and other antimicrobial treatments.^3^ Furthermore, the biofilm structure has been found to facilitate the horizontal transfer of genetic material, contributing to the spread of antibiotic resistance genes.^4^ Moreover, the architecture of biofilms not only allows microbial populations to colonize human and animal hosts but also enables their attachment to inert surfaces. These features have led to an increasing prevalence of infections linked to the use of biomaterials in both human and veterinary medical applications.^5^ Consequently, considerable efforts are underway to develop alternative strategies for managing biofilm-forming MDR infections.^6^

Bacteriophages (in short phages), commonly encountered viruses capable of infecting bacterial cells, offer significant potential in managing biofilm development and bacterial infections.^7–9^ The use of diverse phages, often in the form of a phage cocktail, has been shown to effectively reduce biofilm growth on various surfaces, organic and inorganic alike, making a notable contribution to disease prevention.^8–10^ However, recent studies have shown that biofilm can regrow after 24 hours of phage treatment, which may limit the effectiveness of phage therapy. A study^11–13^ examined the efficacy of phages against biofilms formed by *Escherichia coli, Listeria monocytogenes* and *Pseudomonas aeruginosa*. The researchers discovered that phages significantly reduced the biofilm at 24 hours, but their effectiveness diminished by 48 hours. Other work reports while phages initially reduced the biofilm biomass, after 48 hours the biofilm had returned to its original level.^14^,^15^ A similar observation was reported in the case of phage treatment of *Acinetobacter baumannii* and *P. aeruginosa* biofilms.^16^,^17^ In this case, the biofilm’s bacterial cells developed resistance to the phages over time.

Combining phages with other treatment approaches, such as antibiotics, can potentially enhance therapeutic effects.^18^ To date, much of the research on phage-antibiotic combinations has centered around *P. aeruginosa*, a well-known opportunistic pathogen often linked to cystic fibrosis, burn infections, hospital-acquired pneumonia, and urinary tract infections.^19^

Another pathogenic bacterium of concern is *E. coli*, which is associated with both intestinal and extra-intestinal diseases, such as diarrhea, colitis, urinary tract infections, bacteremia, and sepsis, posing a significant global public health challenge.^20^ Recent studies have demonstrated a phage-antibiotic synergy between phage T4 and variable concentrations of cefotaxime when targeting *E. coli*.^21^

Metal nanoparticles, which consist of a single metallic element or its oxide on the nanometer scale, are another factor that can be utilized in combination with phages. Silver and gold nanoparticles (AgNPs and AuNPs respectively), in particular, are widely used in medicine and industry for antimicrobial therapy.^22–24^ They have shown effectiveness against various MDR bacterial strains, including *E. coli*,^25^,^26^ *P. aeruginosa*,^27^,^28^ *Klebsiella pneumoniae*,^29^ *Staphylococcus aureus, S. epidermidis, A. baumannii*,^30–32^ and also *Bacillus cereus* and *L. monocytogenes*.^32^ Furthermore, they can synergize with antibiotics such as cefotaxime, ceftazidime, meropenem, ciprofloxacin, and gentamicin to eradicate *E. coli* and *K. pneumoniae*.^26^,^28^

The last findings demonstrate that the simultaneous use of bacteriophages alongside antimicrobial agents such as metal nanoparticles can effectively control the growth of pathogenic bacteria.^33–37^ A particularly promising example of this strategy is combining silver nanoparticles with bacteriophage T7. In our previous work, we demonstrated a novel method to control *E. coli* biofilm using T7 phages armed with AgNPs.^38^ This strategy showed significant effectiveness shortly after application, with notable biofilm reduction observed just 6 hours after treatment.^38^ However, it remained unclear how the biofilm would respond to T7Ag-XII over an extended period. Does the biomaterial composed of phages and AgNPs lose its biofilm-eradicating capability after 24 or 48 hours, allowing the bacterial biofilm to regrow?

Therefore, in this short report, we present the results of the activity of T7Ag-XII phages within 24 and 48 hours of action, which is new and distinguishes these studies. Our findings clearly demonstrate that the biomaterial composed of T7 phages armed with AgNPs remains effective against biofilms over an extended period of activity.

## Materials and Methods

### Bacteria and Phages

*Escherichia coli* C600 (Department of Molecular Virology, UW) and BLT5403 (Merck™) strains were used in this study. The phages used were T7 wild-type (ATCC BAA-1025-B2) and engineered T7Ag-XII bacteriophage displaying the RFEHPAVPRTEM peptide (AgNP-binding peptide) within the gp10B protein.^38^ The cultivation and growth of bacteria were conducted using Lysogeny Broth (LB), LB agar (1.5% agar), and Top-Agar media (0.7% agar) at 37°C. For BLT5403, 100 μg/mL of ampicillin was added. The phage titers were analyzed using double-layer agar plates; the bottom layer contained around 25 mL of LB agar (1.5%) and the upper layer contained 4 mL of Top-Agar (0.7%) with 200 μL of overnight *E. coli* culture.

### Chemicals

Silver nanoparticles, 10 nm particle size, 0.02 mg/mL in aqueous buffer (Merck™), Cat.# 730785; Phosphate-Buffered Saline (PBS), MP Biomedicals, Cat.# 2810305; Crystal violet, Sigma-Aldrich, Cat.# V5265-500ML; Ethanol, POCH, Cat.# 396483150; Ampicillin sodium salt, Sigma-Aldrich, Cat.# A9518-5G were used.

### Biofilm Assay

The biofilm biomass after treatment with T7 phages and/or AgNPs was analyzed following the method described by us previously.^38^ An overnight culture of *E. coli* strain C600 was diluted 1:100 in fresh LB broth medium, then aliquoted into 96-well microplates with 200 µL of bacterial culture per well and incubated at 37°C. Next, the 24-hour biofilms were washed 5 times with PBS. After the last washing, 180 µL of PBS was added to each biofilm. Then, 20 µL of various types of T7 phages and/or AgNPs were added. LB lysates of T7 wt and T7Ag-XII phages at an initial concentration of 1×10^10^ pfu/mL and the water suspension of AgNPs at an initial concentration of 0.02 mg/mL were used as controls for the efficacy of the test agents alone. The preparation of T7Ag-XII-AgNPs biomaterial was carried out by mixing phages and AgNPs and incubating for 30 minutes at room temperature (RT). Serial decimal dilutions of phages, AgNPs or biomaterial in PBS were prepared as needed. Negative control biofilms were treated with PBS alone.

Analyses of biofilm were performed 24 and 48 hours after the addition of the test agents. The biofilm biomass was measured using the crystal violet assay. First, LB broth was removed, and all wells were washed twice with PBS. The plates were then placed on a thermoblock at 50°C for 15 minutes to dry. Next, 200 µL of crystal violet (0.1% w/v) was added and incubated for 15 minutes at RT. After incubation, the crystal violet was removed, and all wells were washed twice with deionized water, then allowed to dry at RT for 15 minutes. Finally, 100 µL of ethanol (99.8%) was added to each well, and the absorbance at λ=590 nm was measured using a Tecan Sunrise Reader.

### Statistics

All data displayed in the charts are expressed as mean values from at least three biological replicates, with error bars indicating standard deviations. Statistical significance was determined using a one-way ANOVA test, and the corresponding P-values were provided. All statistical analyses were conducted using GraphPad Prism version 10.2.2.

## Results

T7 phages armed with AgNPs are a novel and effective antibiofilm strategy. We know that such biomaterial exhibits antimicrobial activity after 6 h of treatment.^38^ Because phages can lose their antibiofilm activity over time, leading to the biofilm re-growing to its pre-phage state, it is important to analyze the biomaterial’s effectiveness over a longer period. Therefore, we conducted an experimental analysis of the eradication of *E. coli* biofilms after 24 and 48 hours using wild-type T7 phages, engineered T7Ag-XII phages (displaying the RFEHPAVPRTEM peptide),^38^ AgNPs alone, and biomaterial contained engineered T7Ag-XII phages armed with AgNPs.

**Figure 1:**
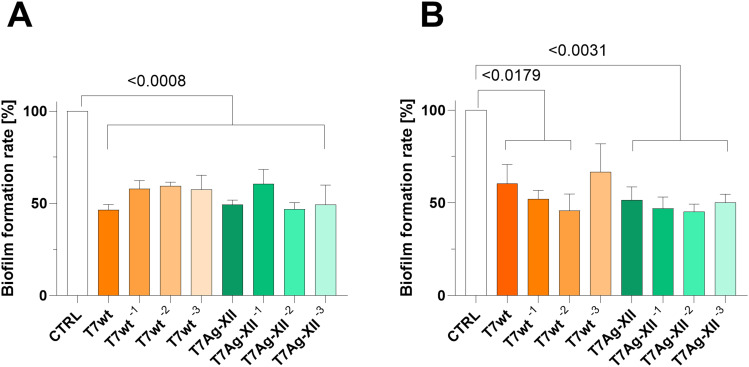
Assessments of biofilm biomass after treatment with T7 wild-type and engineered T7Ag-XII phages after 24 hours (**A**) and 48 hours (**B**) incubation. The initial working concentrations of T7wt and T7Ag-XII phages were kept at 1×10^9^ pfu/mL. The variations in phage working concentrations decreased by an order of magnitude from left to right. Data are shown as mean values of ≥3 biological replicates, and standard deviations are represented by error bars. Statistical analysis (one-way ANOVA) was carried out and significant P-values are presented.

**Abbreviations**: AgNPs, silver nanoparticles; CTRL, biofilm without phages or AgNPs treatment; T7Ag-XII, engineered T7 phages displaying the RFEHPAVPRTEM peptide; T7wt, wild-type T7 phages.

**Figure 2:**
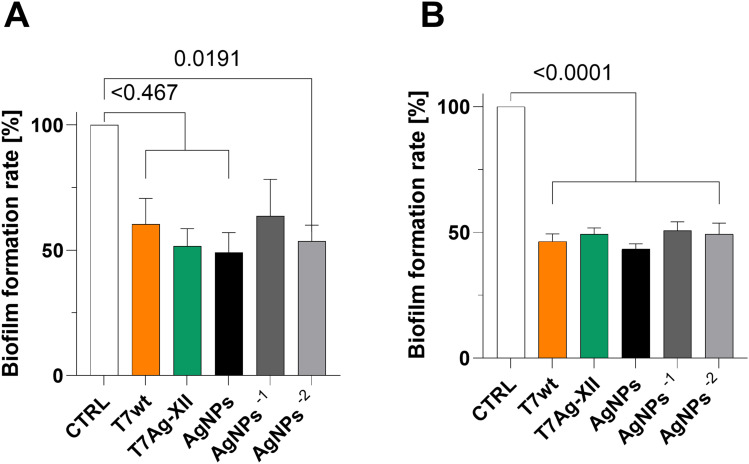
Assessments of biofilm biomass after treatment with T7 phages and AgNPs after 24 hours (**A**) and 48 hours (**B**) incubation. The working concentrations of T7wt and T7Ag-XII phages were kept at 1×10^9^ pfu/mL. The initial working concentration of AgNPs was 0.002 mg/mL and the variations in AgNPs working concentrations decreased by an order of magnitude from left to right. Data are shown as mean values of ≥3 biological replicates, and standard deviations are represented by error bars. Statistical analysis (one-way ANOVA) was carried out and significant P-values are presented.

**Abbreviations**: AgNPs, silver nanoparticles; CTRL, biofilm without phages or AgNPs treatment; T7Ag-XII, engineered T7 phages displaying the RFEHPAVPRTEM peptide; T7wt, wild-type T7 phages.

After 24 and 48 hours of incubation with the higher concentration of either T7 phage type (wt or engineered Ag-XII), the biofilm biomass decreased significantly by approximately 50% (Figure 1). There were almost no significant differences between the phage concentration variants used in the assays. A similar observation was noted after 24 and 48 hours of treatment with various concentrations of AgNPs (Figure 2).

**Figure 3:**
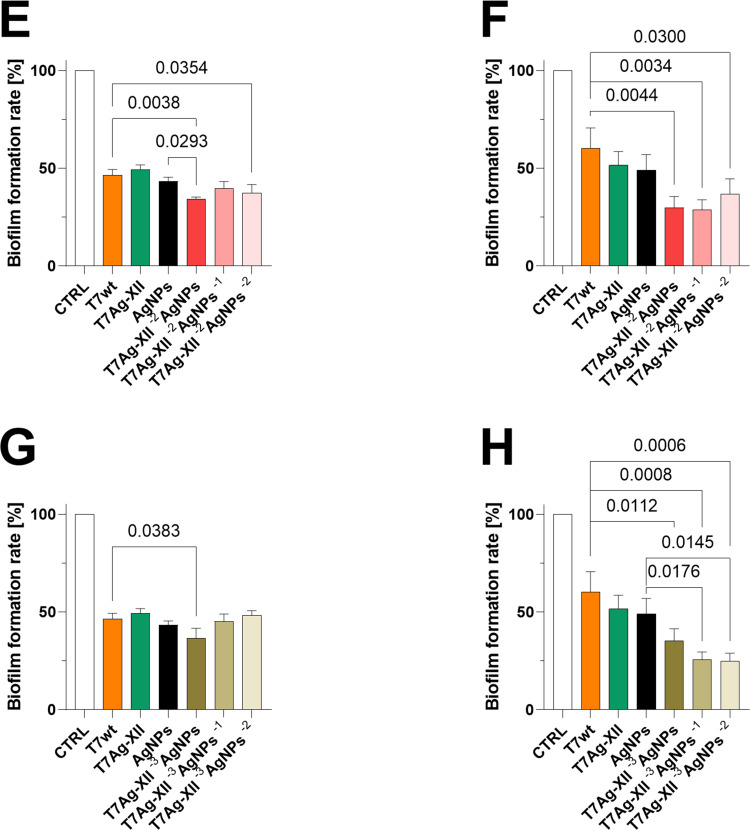
Assessments of biofilms after treatment with T7 phages, AgNPs, or a combination of T7 phages and AgNPs. The working concentrations of T7wt and T7Ag-XII phages were kept at 1×10^9^ pfu/mL (all panels). The working concentration of AgNPs was 0.002 mg/mL (all panels). (**A** and **B**) the working concentration of T7Ag-XII phages in biomaterial was kept at 1×10^9^ pfu/mL and the initial working concentration of AgNPs was 0.002 mg/mL; the variations in AgNP working concentrations decreased by an order of magnitude from left to right. (**C** and **D**) the working concentration of T7Ag-XII phages in biomaterial was kept at 1×10^8^ pfu/mL and the initial working concentration of AgNPs was 0.002 mg/mL; the variations in AgNP working concentrations decreased by an order of magnitude from left to right. (**E** and **F**) the working concentration of T7Ag-XII phages in biomaterial was kept at 1×10^7^ pfu/mL and the initial working concentration of AgNPs was 0.002 mg/mL; the variations in AgNP working concentrations decreased by an order of magnitude from left to right. (**G** and **H**) the working concentration of T7Ag-XII phages in biomaterial was kept at 1×10^6^ pfu/mL and the initial working concentration of AgNPs was 0.002 mg/mL; the variations in AgNP working concentrations decreased by an order of magnitude from left to right. Data are shown as mean values of ≥3 biological replicates, and standard deviations are represented by error bars. Statistical analysis (one-way ANOVA) was carried out and significant P-values are presented.

**Abbreviations**: AgNPs, silver nanoparticles; CTRL, biofilm without phages or AgNPs treatment; T7Ag-XII, T7 phages displaying the RFEHPAVPRTEM peptide; T7wt, wild-type T7 phages.

Notably, the antibiofilm activity of engineered T7Ag-XII phages (at a concentration of 1×10^9^ plaque-forming units [pfu]/mL) combined with various concentrations of AgNPs were significantly higher than the activities of T7wt phages alone or AgNPs alone (Figure 3). Especially, the lower concentrations of engineered T7Ag-XII phages combined with decreasing concentrations of AgNPs characterized with high antibiofilm efficiency after 48 hours of incubation (Figure 3B, D, F, and H). Such a result indicates that the new strategy based on the T7Ag-XII-AgNPs biomaterial is significantly more effective over a longer period than phages alone or nanomaterials alone.
**Figure 3:**
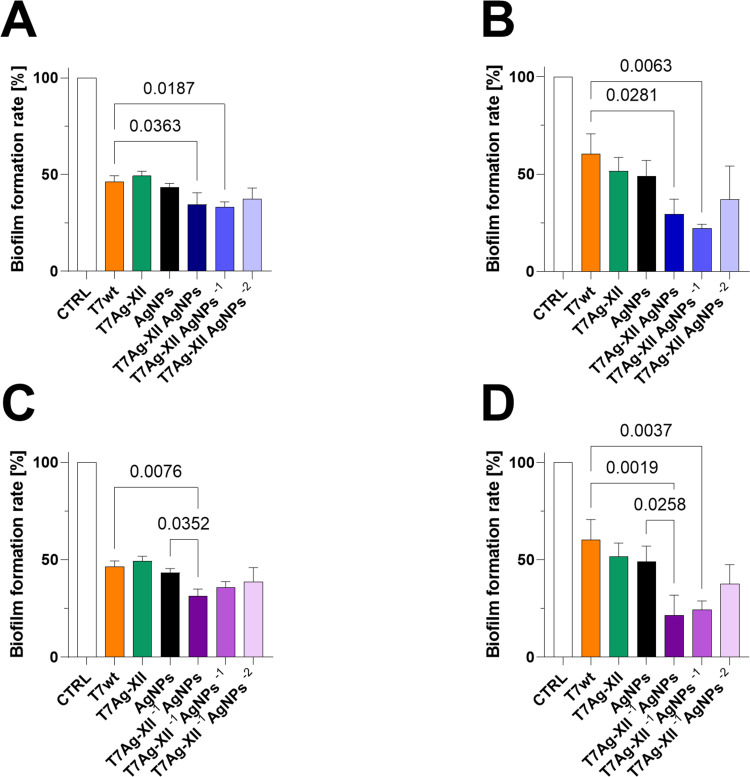
Continued.

## Discussion

For decades bacteriophages have been known as antibacterial agents used to treat bacterial infections, including bacterial biofilms.^39^ They are considered a good alternative to antibiotics, to which pathogenic bacteria are increasingly developing resistance. Despite therapeutic successes, phages also have their weaknesses. They can carry toxins or resistance genes, physically support the biofilm, or induce bacterial resistance to phage infection.^40^ Moreover, there are known cases of regrowth of bacterial biofilms treated with phages. This particularly occurs after prolonged exposure to the phage, which causes the bacteria forming the biofilm to become resistant to phage infection. Several examples described of *E. coli* biofilm acquiring resistance to infection by both a single phage and a phage cocktail.^41–45^ Other types of biofilm-forming bacteria, such as *S. aureus, P. aeruginosa, Salmonella enterica,* and *Serratia marcescens*, also become resistant to phage infection after prolonged exposure.^46–49^ Therefore, in novel alternative therapeutic approaches, phages are used combined with other antimicrobial agents (eg, antibiotics or metal nanoparticles) to improve the efficacy of phage therapy.^33^,^50^

The combination of phages and silver nanoparticles appears to be an intriguing new strategy for combating bacterial biofilms.^33–37^ However, it should be noted that high concentrations of nanoparticles, such as AgNPs, can be toxic to humans.^51^ In our previous work using T7 phages armed with AgNPs to combat *E. coli* biofilm, we demonstrated a potential solution to this problem. We showed that lower concentrations of AgNPs used in the experimental setup were highly effective in eradicating biofilms and were not toxic to eukaryotic cells even after 72 hours of exposure.^38^

The effectiveness of the engineered T7Ag-XII phages armed with AgNPs has so far been studied by us only after a short exposure time of bacterial biofilm to the biomaterial. After 6 hours of action, the T7-AgNPs biomaterial was more effective than the T7 phages or AgNPs alone.^38^ However, it was unclear whether the biomaterial remained effective over a longer time of action. It was particularly interesting to analyze the anti-biofilm effectiveness of the biomaterial after 48 hours, a period during which biofilms often develop resistance to phages and begin to regrow.^11^,^14^,^15^

In this study, we demonstrated a statistically significant increase in biofilm eradication effectiveness by the engineered T7Ag-XII-AgNPs biomaterial. The effectiveness, compared to phages alone and AgNPs alone, was significantly higher, especially after 48 hours of treatment. Contrary to reports describing biofilm regrowth after 48 hours, our new strategy based on T7 phages armed with AgNPs showed increased anti-biofilm effectiveness over time, particularly after 48 hours of incubation. Furthermore, the highest activity was observed when lower concentrations of AgNPs were used for preparing of biomaterial, which positively correlates with previous reports describing similar observations.^38^,^52^

Our work demonstrates that it is possible to enhance the natural enemies of bacteria - bacteriophages - by arming them with additional antibacterial agents such as silver nanoparticles. We believe that our research will contribute to broader interest in improving the therapeutic properties of phages, thereby increasing the effectiveness of phage therapy.

## Conclusion

In this work, we explored the enhanced antibacterial potential of T7 phages armed with silver nanoparticles as a strategy for combating bacterial biofilms. While phages are a promising alternative to antibiotics, bacteria can develop resistance to phage treatment, particularly in biofilms. By combining T7 phages with AgNPs, we observed a significant increase in biofilm eradication, particularly after 48 hours, a time frame where biofilms typically become resistant. Importantly, lower concentrations of AgNPs were more effective and non-toxic to eukaryotic cells, offering a promising solution for improving phage therapy without harmful side effects. We believe that our work demonstrates the potential to enhance phage therapy, leading to more effective treatment of bacterial infections, particularly those involving biofilm formation.
